# Abnormal Changes of Brain Cortical Anatomy and the Association with Plasma MicroRNA107 Level in Amnestic Mild Cognitive Impairment

**DOI:** 10.3389/fnagi.2016.00112

**Published:** 2016-05-18

**Authors:** Tao Wang, Feng Shi, Yan Jin, Weixiong Jiang, Dinggang Shen, Shifu Xiao

**Affiliations:** ^1^Department of Geriatric Psychiatry, Shanghai Mental Health Center, Shanghai Jiao Tong University School of MedicineShanghai, China; ^2^Alzheimer’s Disease and Related Disorders Center, Shanghai Jiao Tong UniversityShanghai, China; ^3^IDEA Lab, Department of Radiology and BRIC, University of North Carolina at Chapel HillChapel Hill, NC, USA; ^4^Department of Brain and Cognitive Engineering, Korea UniversitySeoul, South Korea

**Keywords:** Alzheimer’s disease, amnestic mild cognitive impairment, genetics, biological markers, surface-based morphometry

## Abstract

MicroRNA107 (Mir107) has been thought to relate to the brain structure phenotype of Alzheimer’s disease. In this study, we evaluated the cortical anatomy in amnestic mild cognitive impairment (aMCI) and the relation between cortical anatomy and plasma levels of Mir107 and beta-site amyloid precursor protein (APP) cleaving enzyme 1 (BACE1). Twenty aMCI (20 aMCI) and 24 cognitively normal control (NC) subjects were recruited, and T1-weighted MR images were acquired. Cortical anatomical measurements, including cortical thickness (CT), surface area (SA), and local gyrification index (LGI), were assessed. Quantitative RT-PCR was used to examine plasma expression of Mir107, BACE1 mRNA. Thinner cortex was found in aMCI in areas associated with episodic memory and language, but with thicker cortex in other areas. SA decreased in aMCI in the areas associated with working memory and emotion. LGI showed a significant reduction in aMCI in the areas involved in language function. Changes in Mir107 and BACE1 messenger RNA plasma expression were correlated with changes in CT and SA. We found alterations in key left brain regions associated with memory, language, and emotion in aMCI that were significantly correlated with plasma expression of Mir107 and BACE1 mRNA. This combination study of brain anatomical alterations and gene information may shed lights on our understanding of the pathology of AD.

Clinical Trial Registration: http://www.ClinicalTrials.gov, identifier NCT01819545.

## Introduction

Alzheimer’s disease (AD) is characterized by a progressive decline in cognition and daily function. It is the most common form of dementia worldwide. Pathologically, AD is defined by the intracellular accumulation of aggregated and hyperphosphorylated tau protein, the extracellular deposition of amyloid β (Aβ) peptides, and the accumulation of neurofibrillary tangles and amyloid plaques throughout the cortex (De Strooper, 2010; Kandimalla et al., 2013a,b). Amnestic mild cognitive impairment (aMCI) is thought to be a predementia phase of AD (Albert et al., 2011), and is characterized by the same etiology to a lesser degree (Petersen et al., 2006). A widely accepted hypothesis links the major pathology of AD to the generation and subsequent accumulation of Aβ through sequential cleavage of amyloid precursor protein (APP) by beta-site APP cleaving enzyme 1 (BACE1)^1^ and γ-secretase (Dislich and Lichtenthaler, 2012). Regulation of expression of the proteins involved in this process plays an important role in AD (Bettens et al., 2009).

BACE1 expression is regulated by BACE1 messenger RNA (BACE1 mRNA), while BACE1 mRNA is regulated by microRNA (Mir/miRNA). Accumulating evidence suggests that alterations in Mirs contribute to AD (Sathya et al., 2012). Studies have found decreases in Mirs in postmortem brain of AD patients, as compared to normal controls (NCs). More interestingly, Mir expressions are altered not only in the brains of AD patients, but also in their cerebral spinal fluid (CSF) and blood plasma (Hébert et al., 2010). Among these, MicroRNA107 (Mir107)^2^ targets genes directly related to AD, including BACE1. In microarray studies, Mir107 levels were substantially reduced in the temporal cortex of AD patients (Wang et al., 2011).

The accumulation of neurofibrillary tangles and amyloid plaques in the cortex is associated with gray matter (GM) atrophy and volume decline. Abnormal brain anatomy has been identified as an important feature of the pathophysiological process of AD, and can be visualized using different modalities of MRI for GM (Meda et al., 2013) and white matter (Li et al., 2013; Jin et al., 2015; Wang et al., 2016), respectively. Those features can be accurately used to differentiate AD patients from the normally aging population (Zhan et al., 2014, 2015). Morphological parameters have been widely used to detect brain abnormalities in aMCI. Previous voxel-based morphometry (VBM) studies reported that aMCI subjects showed GM atrophy in entorhinal cortex, posterior cingulate cortex, and medial prefrontal cortex (Apostolova et al., 2007). Although VBM is valuable in measuring morphological changes in AD patients, it does not capture cortical sulcal and gyral patterns, or their changes due to the disease (Davatzikos, 2004).

Surface-based morphometry (SBM) is one approach that captures subtle cortical surface changes, and can examine cortical thickness (CT) and surface area (SA) of GM separately. Studies have reported that aMCI patients showed overall cortical thinning and sulcal widening, compared to NCs (Davatzikos, 2004). SBM can also assess the degree of cortical folding using local gyrification index (LGI; Im et al., 2008; Li et al., 2014a). These measurements provide unique and complementary information that reflects distinct cortical properties (Li et al., 2014b; Libero et al., 2014). Investigation of these surface-based measures can provide information beyond those volumetric abnormalities previously uncovered in AD. By measuring CT, SA, and LGI, we may uncover the underlying changes in cortical architecture of AD (Libero et al., 2014).

A definite diagnosis of AD is determined by postmortem examination. Currently, amyloid PET examination (Fleisher et al., 2012) can be utilized for early diagnosis, but, because PET examination is expensive, a routine diagnosis of aMCI and AD still depends on combination of clinical and neuropsychological tests. Therefore, there is great interest in identifying AD genotype and phenotype associated biomarkers in brain anatomy and plasma.

Our goal in this study is to investigate alterations in cortical anatomy in aMCI, as well as to detect any relationship between structural changes and neuropsychological scores, levels of Mir107, and BACE1 mRNA in plasma. The findings of this study will provide valuable information about the abnormal neuroanatomy of aMCI, and also highlight a potential connection between structural changes and plasma Mir107 and BACE1 mRNA.

## Materials and Methods

### Participants

This study was registered as a clinical trial with ClinicalTrials.gov registry number as NCT01819545^3^. We recruited 20 patients with aMCI from the Shanghai Mental Health Center, Shanghai Jiao Tong University School of Medicine, and 24 cognitively normal elderly subjects from the Shanghai Changning District. Patients were enrolled by the hospital via self-referral or the referral from family or physician. This study was approved by the Institution’s Ethical Committee of Shanghai Mental Health Center, Shanghai Jiao Tong University School of Medicine, and written informed consent was obtained from all subjects and/or their legal guardians. All experiments were performed in accordance with relevant guidelines and regulations.

aMCI was diagnosed based on the previously published criteria (McKhann et al., 2011). We amended the aMCI diagnostic criteria of the Petersen Mini Mental States Examination (MMSE; Folstein et al., 1975) in order to accommodate the low level of education in elderly Chinese. In the current study, we used revised MMSE cut-off scores as one of the criteria to recruit individual subjects (Katzman et al., 1988). The cognitively normal elderly control subjects (NC) were also recruited. They were independently functioning community dwellers with no neurological or psychiatric conditions.

All participants underwent a screening process that included a review of their medical history, physical and neurological examinations, laboratory tests, and MRI scans. The clinical assessment of MCI or dementia included neuropsychological tests, as well as behavioral and psychiatric interviews conducted by the attending psychiatrists. MMSE and Montreal Cognitive Assessment (MoCA; Nasreddine et al., 2005) were assessed in all participants. Based on assessment, aMCI patients were retained, and others who had impairments in a single non-memory domain or impairment in two or more domains were excluded.

### MR Image Acquisition and Processing

MRI images were scanned with a Siemens MAGNETOM VERIO 3T scanner (Siemens, Munich). T1-weighted images were obtained with 128 sagittal slices using the 3D magnetization prepared rapid acquisition gradient echo sequence with the following parameters: TR = 2530 ms, TE = 3.39 ms, flip angle = 7°, spatial resolution = 1 × 1 × 1.3 mm^3^, and the acquisition time was 8 min 7 s. The MRI FLAIR data acquisition setting used the following parameters: matrix 256 × 192, NEX = 1, FOV = 24 cm, TE = 140, TR = 8600, InVTime = 2200.

Surface anatomy was extracted from MR images using FreeSurfer software package (version 5.3.0)^4^ (Fischl and Dale, 2000). We reviewed all obtained cortical surfaces and minimal manual editing was also performed at inaccurately segmented locations. The generated cortical surfaces were validated by comparing them with manual measures on MRI data (Fischl and Dale, 2000). CT was computed as the average distance between gray-white surface and pial surface. SA for each vertex was calculated on the pial surface, representing the area of the tessellated triangles linked to the vertex. The local cortical folding for each vertex was measured with LGI, which accounted for the ratio of local SA to the outer hull layer that tightly wrapped the pial surface. The folding was extended from two-dimensional gyrification measurement (Schaer et al., 2008).

### Blood Plasma Collection

Blood samples were obtained from each subject and were centrifuged for 20 min at 4°C at 3000 rpm. A 200 μl plasma aliquot was taken from each sample and immediately frozen and stored at −80°C.

### Quantitative RT-PCR (qRT-PCR)

Total mRNA and miRNA were extracted using TRIZOL^®^ Reagent (Invitrogen, Carlsbad, CA, USA), and were quantified using a NanoDrop^®^ ND-1000 spectrophotometer (Waltham, MA, USA). Total mRNA was subjected to qRT-PCR using 2× PCR master mix (Super Array, Valencia, CA, USA) and the ABI PRISM7900 system (Applied Biosystems, Foster City, CA, USA). For each sample, real-time PCR was performed for the target mRNA (BACE1; Table 1) together with the reference gene β-actin. The relative expression of the target mRNA was determined by the 2^ΔΔCT^ method.

**Table 1:** **The sequence of primers used for the RT-PCR of BACE1 mRNA and Mir107**.

Gene	Primer sequence	Annealing temperature (°C)	Lengths of PCR products (bp)
BACE1	F:5′AAGTTCATTACCTCCCTATCAGT3′	60	186
mRNA	R:5′AGGCCCTCCTTGTATTTCC3′
Mir107	GSP:5′GCAGCAGCATTGTACAGG3′	60	65
	R:5′CAGTGCGTGTCGTGGAGT3′		

Total miRNA qRT-PCR was conducted using the Universal cDNA Synthesis Kit (Exiqon, Vedbaek, Denmark) and the Gene Amp PCR System 9700 (Applied Biosystems, Foster City, CA, USA). Similarly, for each sample, real-time PCR was performed for the target Mir107 (Table 1) together with the reference gene microRNA423–5p. The relative expression of the target miRNA was determined by the 2^ΔΔCT^ method.

### BACE1 Protein Concentration

We used a BACE1 protein ELISA kits (LBL^®^ 27752 human BACE1 assay kit, Takasaki-Shi, Gunma, Japan) to assay the BACE1 protein concentration from our study participants.

### Statistical Analysis

SPSS 17.0 was used for statistical analysis. Two-tailed *t*-tests were used to compare demographic characteristics between groups. Chi-square tests were used to measure differences in gender distribution. For each vertex, a general linear model was used to detect significant differences in CT, SA, and LGI between aMCI and controls patients, respectively. The confounding factors were regressed, including age, gender, education, and overall measurement. A smoothing kernel of 10 mm was applied before group comparison at the level of each vertex. Mir107, BACE1 mRNA, and BACE1 protein expression between groups were analyzed by two-tailed *t*-tests. Pearson correlation was used to examine correlations between plasma Mir107 and BACE1 mRNA expression and cortical anatomy.

## Results

### Demographic and Clinical Variables

The demography and clinical scores for the aMCI group and the NC group are listed in Table 2. No significant differences between the groups were observed in age, gender or education, so the effects of age, gender, education level, and brain size were removed in our analysis. As expected, there were group differences in the MMSE, MoCA and Clinical Dementia Rating-Sum of Box (CDR-SOB) scores.

**Table 2:** **Demography, clinical scores, RT-PCR of Mir107, BACE1 mRNA, and protein expression in plasma of the subjects in the study**.

					95% CI of the Mean difference	
	aMCI (*n* = 20)	NC (*n* = 24)	*p*-value	Mean difference	Lower	Upper
Age (years)	70.1 ± 7.2	69.9 ± 7.6	0.81	0.18	−4.34	4.7
Male/Female	9/11	13/11	0.55	–	–	
Education (years)	7.8 ± 4.9	9.4 ± 4.1	0.91	−1.58	−4.32	1.17
MMSE	24.5 ± 3.4	27.8 ± 2.8	<0.001	−3.25	−4.96	−3.25
MoCA	18.3 ± 4.3	24.0 ± 3.6	<0.001	−5.73	−8.2	−3.25
CDR-SOB	2.0 ± 0.7	0.0 ± 0.0	<0.001	2	1.67	2.34
Mir107^1^	2.33 ± 2.24	3.85 ± 1.24	0.007	−1.52	−2.6	−0.44
BACE1mRNA^2^	1.25 ± 0.50	0.88 ± 0.37	0.007	0.37	0.11	0.63
BACE1 (LINE)	3.95 ± 1.82	3.22 ± 1.07	0.106	0.73	−0.16	1.62
BACE1 (LOG)	4.91 ± 1.85	4.17 ± 1.25	0.122	0.74	−0.21	1.69
*r* (1 and 2)	−0.761	−0.648	–	–	–	–
*p*-value (1 and 2)	<0.001	0.001	–	–	–	–

*Data represent mean ± SD and were analyzed by t-test; the p-values were determined from two-tailed t-tests except for gender, which was analyzed using the Chi squared test; 1 = Mir107, 2 = BACE1 mRNA, Pearson Correlation analysis; Symbol: – denotes Not Applicable*.

### Mir107, BACE1 mRNA, and Protein Level in Plasma

Plasma levels of Mir107 and BACE1 mRNA were significantly different between aMCI and NC (Table 2). There were significant negative correlations between plasma Mir107 and BACE1 mRNA gene expression in aMCI and NC (Table 2). We did not find any significant difference in the plasma level of BACE1 protein between aMCI and NC subjects (Table 2).

### Cortical Thickness

**Figure 1:**
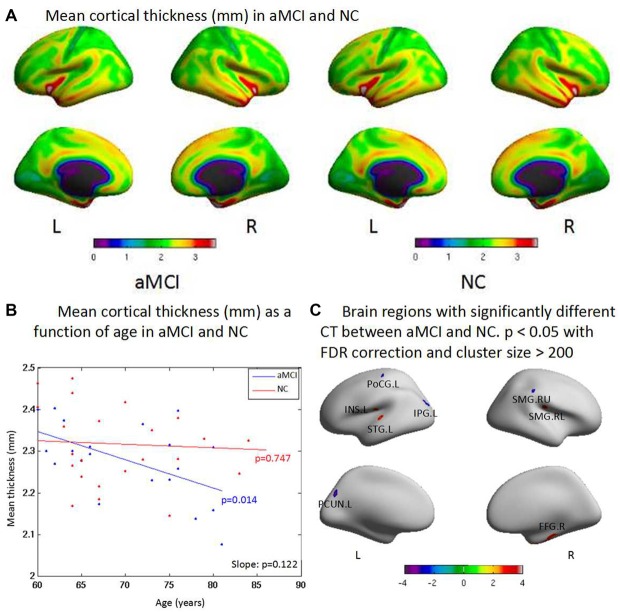
**(A)** The cortex thicknesses are thinner in the superior parietal gyrus, postcentral gyrus, lingual gyrus and paracentral gyrus than other brain regions both in amnestic mild cognitive impairment (aMCI) and normal control (NC) in general. **(B)** aMCI shows less cortical thickness (CT) than NC when age increases but no significant difference is found between groups (*p* = 0.122). There is significant difference within group of aMCI when age increases (*p* = 0.014), while there is no significant difference within group of NC when age increases (*p* = 0.747). **(C)** The ROI-based analysis of CT revealed that the left postcentral gyrus (PoCG.L), the left inferior parietal gyrus (IPG.L), the left precuneus (PCUN.L) and the upside right supramarginal gyrus (SMG.RU) were significant group differences (*p* < 0.05) between the aMCI and the NC groups with the aMCI having thinner cortex than the NC. In addition, the left superior temporal gyrus (STG.L), the left insula (INS.L), the low side right supramarginal gyrus (SMG.RL) and the right fusiform gyrus (FFG.R) exhibited significantly (*p* < 0.05) larger thickness in the aMCI compared with the NC.

Samples from patients with aMCI showed widespread thinning of CT as compared to NC in the memory-associated cortical areas. CT was thinner in the superior parietal gyrus, postcentral gyrus, lingual gyrus, and paracentral gyrus, compared to other brain regions both in aMCI and NC subjects (Figure 1A). There was significant difference within the aMCI patients with increased age (*p* = 0.014), but similar changes were not seen in the NC subjects (Figure 1B). Left brain areas associated with episodic memory and language showed the largest differences (*p* < 0.05) between the groups in left postcentral gyrus, left inferior parietal gyrus, left precuneus, and the upside right supramarginal gyrus. In addition, the left superior temporal gyrus and insula, as well as the low side right supramarginal fusiform gyrus, were significantly thicker in aMCI (*p* < 0.05, Figure 1C).

### Cortical Surface Area

**Figure 2:**
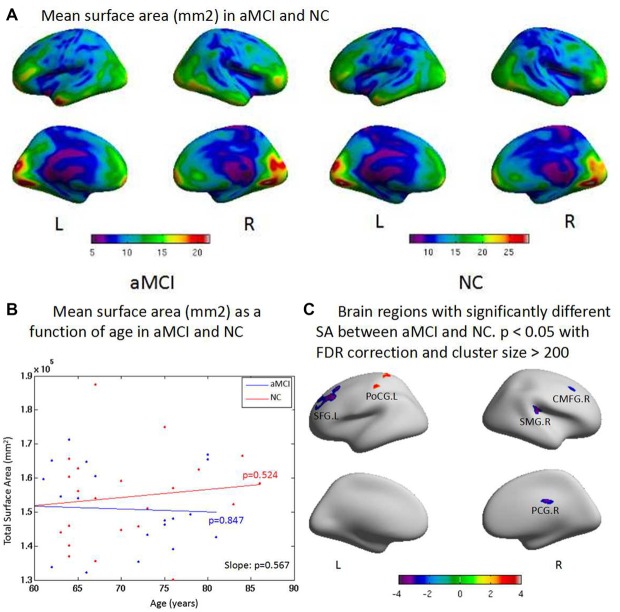
**(A)** There is no significant difference between two groups on the total surface area (SA). But aMCI shows less SA than NC when age increases. The surface areas are less in the superior parietal gyrus, postcentral gyrus, middle temporal gyrus and anterior cingulate gyrus than other brain regions both in aMCI and NC in general. **(B)** aMCI shows less SA than NC when age increases but no significant difference is found between groups (*p* = 0.567). There are no significant difference within group of aMCI (*p* = 0.847) and NC (*p* = 0.524) when age increases, respectively. **(C)** ROI based group analysis found significantly (*p* < 0.05) smaller SA in the left superior frontal gyrus (SFG.L), the right supramarginal gyrus (SMG.R), the right caudal middle frontal gyrus (CMFG.R) and the right posterior cingulate gyrus (PCG.R) in the aMCI, compared with the NC. In addition, the left postcentral gyrus (PoCG.L; *p* < 0.01) exhibited significantly larger SA in the aMCI compared with the NC.

There was no significant change between two groups in total SA, but aMCI patients showed reduced SA with the increased age when compared to NC. SA was reduced in the superior parietal gyrus, postcentral gyrus, middle temporal gyrus, and anterior cingulate gyrus in general (Figure 2A). There was no significant difference between aMCI and NC with the increased age (Figure 2B). ROI-based group analysis found the significantly reduced SA in left superior frontal gyrus, right supramarginal and caudal middle frontal gyrus, and right posterior cingulate gyrus in aMCI (*p* < 0.05). In addition, left postcentral gyrus had the significantly larger SA in aMCI (*p* < 0.01, Figure 2C).

### Local Gyrification Index

**Figure 3:**
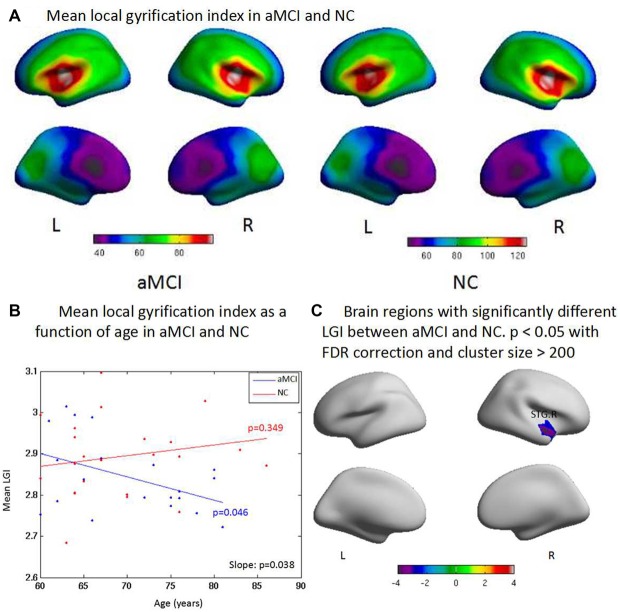
**(A)** Compared with controls, aMCI has significant difference on the general local gyrification index (LGI) and one cluster where aMCI has significant LGI reduction. The LGI are higher in the superior temporal gyrus, and middle temporal gyrus than other brain regions both in aMCI and NC in general. **(B)** aMCI shows less CT than NC when age increases and significant difference is found between groups (*p* = 0.038). There is significant difference within group of aMCI when age increases (*p* = 0.046), while there is no significant difference within group of NC when age increases (*p* = 0.349). **(C)** The ROI based LGI group analysis shows a significant effect of the right superior temporal gyrus (STG.R; *p* < 0.05) in aMCI.

The aMCI group had significantly lower LGI in general. LGI was higher in the superior temporal gyrus and middle temporal gyrus than other brain regions in both groups (Figure 3A). aMCI showed reduced LGI with increase of age; a significant difference was found between groups (*p* = 0.038). There was also a significant decrease with the increased age within aMCI (*p* = 0.046, Figure 3B). The ROI-based LGI group analysis showed a significant effect in the right superior temporal gyrus (*p* < 0.05) in aMCI (Figure 3C).

### Abnormal Surface Anatomy, Neuropsychological Scores, and Plasma Mir107 and BACE1

**Figure 4:**
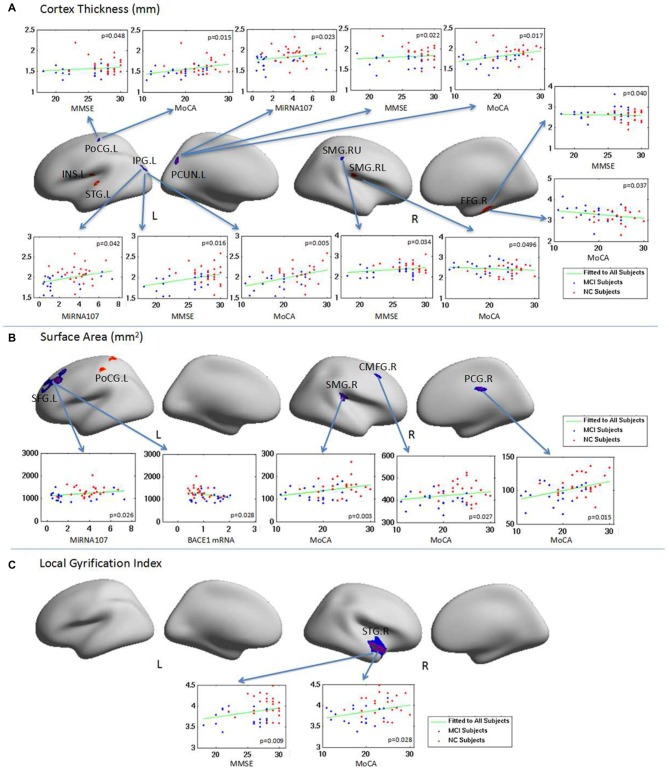
**The relationship between brain regions with abnormal surface anatomy in aMCI and neuropsychological test scores and the plasma target gene expressions.** Changes in CT, SA and LGI in aMCI were correlated with neuropsychological scores and/or plasma MiRNA 107 expression. **(A)** The relationship of CT with neuropsycological test score and the plasma expression of MiRNA 107. **(B)** The relationship of SA with neuropsycological test score and the plasma expression of MiRNA 107 and BACE1 mRNA. **(C)** The relationship of local gyrification and neuropsycological test score.

Changes in CT in aMCI were correlated with neuropsychological scores and plasma Mir107 expression (Figure 4A). Left postcentral gyrus CT was correlated with MMSE and MoCA. Left inferior parietal gyrus and precuneus cortex CT were correlated with MMSE and MoCA, as well as plasma Mir107. Right superior supramarginal gyrus CT was correlated with MMSE, while the right inferior supramarginal gyrus was negatively correlated with MoCA. Right fusiform gyrus CT was negatively correlated with MMSE and MoCA (Figure 4A).

Changes in aMCI SA were also correlated with neuropsychological test scores and plasma Mir107 and BACE1 mRNA expression (Figure 4B). SA of the left superior frontal gyrus was positively correlated with plasma Mir107 expression and negatively correlated to plasma BACE1 mRNA expression. SA of the right supramarginal gyrus, right caudal middle frontal gyrus, and right posterior cingulate cortex were positively correlated to MoCA (Figure 4B). We also found significant reduction in LGI in the right superior temporal gyrus in aMCI, which was correlated with MMSE (*p* = 0.009) and MoCA (*p* = 0.028, Figure 4C).

## Discussion

The main goal of this study is to investigate changes in cortical anatomy in aMCI patients, especially on CT, SA, and LGI, and also to determine any correlation between structural changes and Mir107 and BACE1 plasma levels. The results obtained demonstrate a correlation between *CTs of left precuneus cortex and left inferior parietal gyrus* and plasma levels of Mir107, and between *SA of left superior frontal gyrus* and plasma levels of Mir107 and BACE1 mRNA. We hypothesize that the left precuneus cortex, left inferior parietal gyrus, and left superior frontal gyrus are the areas first damaged in aMCI, and form the core regions of AD impairment. We also hypothesize that Mir107 is critical to the structural changes in AD brain cortex that are associated with cognition, language, and emotion.

### Alterations in Cortical Thickness

We found that CT, especially the CT of left heteromodal association, was thinner in aMCI patients than in NC. CT is impacted by proliferation of myelin, a reduction in neuronal size or number, and changes in synapses (Sowell et al., 2004). CT is also influenced by the number and size of cells within a column, packing density, and by the extent of myelination (Eickhoff et al., 2005). Thus, alterations in CT may reflect abnormality in the underlying cell counts and organization.

Decreased CT in the left postcentral gyrus, left inferior parietal gyrus, and left precuneus cortex found in this study is in line with previous studies of aMCI, in which late aMCI showed cortical thinning in the bilateral dorsolateral prefrontal, anterior and medial temporal, and temperoparietal association cortices, as well as the precuneus (Ye et al., 2014). We found increased cortex thickness in the left superior temporal gyrus cortex, left insula, low side right supramarginal gyrus, and right fusiform gyrus in aMCI. These areas are associated with social cognition processes and word recognition. Some compensations probably occur in the onset of early AD stage.

Some CT results suggest that thinning of heteromodal association cortices is an early biomarker of AD, not hippocampal loss (Sabuncu et al., 2011). We did not find significant hippocampal volume loss in aMCI; instead, we found changes in the heteromodal association cortices, such as left inferior parietal gyrus and left precuneus cortex. Researchers have also reported that AD patients had the reduced CT bilaterally in the precuneus, which is characteristic of AD pathology (Watson et al., 2015). The precuneus is important for the recall of episodic memories, and is involved in source memory with the left inferior prefrontal cortex (Sadigh-Eteghad et al., 2014). The role of the precuneus is suggested to provide the rich episodic contextual associations used by the prefrontal gyrus to select correct memories (Sadigh-Eteghad et al., 2014). In recollection, the precuneus seems to determine whether context information exists, and therefore could be involved in diverse processes as episodic memory retrieval, working memory, and conscious perception.

Our findings are consistent with the Pittsburgh Compound-B (PIB) and Fluorodeoxyglucose—Positron Emission Tomography (FDG-PET) imaging studies. For example, AD patients showed significant PIB retention in the bilateral precuneus, temporal lobe, and cingulate gyrus, as well as glucose hypometabolism in these areas as well as the inferior parietal and middle frontal gyrus, left precentral and parahippocampal gyrus, right superior frontal gyrus, and thalamus (He et al., 2015). The results seen in our study are in line with previous studies of aMCI. We conclude from these findings that structural changes may occur at the onset of aMCI, and can be detected by CT assessment.

### Alterations in Surface Area

We found reduced SA in the left superior frontal gyrus, right supramarginal gyrus, right caudal middle frontal and right posterior cingulate gyrus in aMCI. A few prior studies have examined SA in aMCI with rather inconsistent results. The SA regions implicated in aMCI and NC include the superior temporal gyrus, lingual gyrus, and superior frontal gyrus in the left hemisphere, as well as the angular gyrus, lateral orbitofrontal sulcus, inferior parietal sulcus, middle frontal rostral region, pars triangularis gyrus, central sulcus, temporal pole, superior temporal sulcus, and the precentral gyrus in the right hemispheres (Li et al., 2014b). Another study found the SA in progressive aMCI was slightly greater than in AD (Liao et al., 2014), but with lower cortical GM surface areas in left superior temporal, supramarginal, and inferior parietal cortices (Madsen et al., 2015).

SA has been previously found to be strongly positively correlated with head size and brain size (Dickerson et al., 2009), and is also related to the number of minicolumns in cortex, as the SA of an area is driven by the number of columns. This is important considering the increased number of minicolumns in the frontal and temporal areas in aMCI (Casanova et al., 2010). Thus, alterations in SA in aMCI may be explained by abnormal minicolumn counts, or overall differences in brain size.

### Alterations in Cortical Folding

Sulcal folds are the principal surface landmarks of the cerebral cortex, and exhibit structurally complex patterns that are postulated to reflect underlying connectivity (Van Essen, 1997). Changes in folding geometry have been shown to develop with aging and to be associated with cognitive decline (Kochunov et al., 2005; Liu et al., 2011). Sulcal of individuals with MCI and AD had less curvature and depth than those of cognitively NCs (Im et al., 2008). These differences were observed to be the largest in the temporal lobe. Cognitive functioning, as assessed by MMSE scores, decreased as global cortex gyrification decreased. Abnormalities of global cortex gyrification and regional sulcal span are characteristic of patients with even mild AD and they may thus facilitate early diagnosis of this condition (Liu et al., 2012).

There are reports of mild AD patients with lower gyrification index than controls (King et al., 2010). A consistent pattern of gyrification changes was seen also in dementia subjects, with regions generally affected early in the progression of AD pathology for decreased gyrification (Lebed et al., 2013). We found a reduction in global gyrification in aMCI. A previous study found sulcal widening in AD in the frontal, parietal, temporal, and occipital lobes (Im et al., 2008). Our present study found AD-associated widening of individual sulcal within brain lobes, particularly in the superior frontal and superior temporal sulcal. We suspect the right superior temporal gyrus may be a contributor to the AD effects observed. The gyrification of right superior temporal gyrus in the controls and aMCI patients was reported to be positively associated with MMSE and MoCA scores, supporting previous associations between cognitive performance and cortical morphology in elderly individuals (Liu et al., 2011).

### Plasma Levels of Mir107 and BACE1 mRNA and Abnormal Cortical Anatomy

There was a significant change in Mir107 and BACE1 mRNA in the plasma samples from aMCI and NC subjects, suggesting that plasma Mir107 and BACE1 mRNA expression may be linked to the decrease in CT in the precuneus and cortical SA of the left superior frontal gyrus. Evidence has suggested that the alterations of miRNAs could contribute to AD risk (Hébert et al., 2010). The study has demonstrated that specific miRNA levels were altered in the brains, CSF, and plasma of AD patients (Hébert et al., 2010), raising the possibility of using these compartments for diagnosis.

A combination of genetic data and neuroimaging techniques is increasingly becoming a winning strategy in identifying preclinical stages of AD. By MRI, FDG-PET, and amyloid PET, characteristics of AD can be detected in clinically normal subjects who are genetically predisposed to the disease. In particular, presenilin-1 mutation carriers with aMCI have smaller brain volumes in the thalamus, splenium and pons, and originally involve with the left temporal lobe (Lee et al., 2013). Apolipoprotein E genotype and family history also have an independent and/or additive contribution to brain structural changes (Honea et al., 2010).

In this study, we demonstrate for that peripheral Mir107 and BACE1 mRNA levels were associated with abnormal brain cortical anatomy, including the decrease in CT of precuneus cortex and in SA of left superior frontal gyrus in aMCI patients. These finding suggest that peripheral Mir107 and BACE1 mRNA can be a candidate of early biomarkers for AD and may be important for the onset of AD. We also found that the plasma level of Mir107 was altered in the same fashion as plasma BACE1 mRNA, suggesting that Mir107 may be central in the pathogenesis of AD. This combination study of brain anatomical disruption and gene information, to our knowledge, has not been attempted previously and may shed light on our understanding of pathology of AD beyond amyloid protein theory. In the future, we will continue acquiring MRI scans with more subjects and will conduct a larger population study to further explore this topic.

## Author Contributions

TW, SX and DS designed the study. SX and DS were responsible for the study, supervised data collection, statistical analysis, and modified the manuscript. TW, FS and YJ carried out statistical analysis, and wrote the main manuscript text. TW, FS, YJ and WJ prepared Figures 1–4. All authors reviewed the manuscript.

## Conflict of Interest Statement

The authors declare that the research was conducted in the absence of any commercial or financial relationships that could be construed as a potential conflict of interest.

## Footnotes

^1^http://www.informatics.jax.org/marker/MGI:1346542

^2^http://www.informatics.jax.org/marker/MGI:3619063

^3^www.clinicaltrials.gov

^4^http://surfer.nmr.mgh.harvard.edu/
